# Preliminary Automated Determination of Edibility of Alternative Foods: Non-Targeted Screening for Toxins in Red Maple Leaf Concentrate

**DOI:** 10.3390/plants8050110

**Published:** 2019-04-26

**Authors:** Joshua M. Pearce, Maryam Khaksari, David Denkenberger

**Affiliations:** 1 Department of Material Science and Engineering and Department of Electrical and Computer Engineering, Michigan Technological University, Houghton, MI 49931, USA; 2 Department of Electronics and Nanoengineering, School of Electrical Engineering, Aalto University, FI-00076 Espoo, Finland; 3 Chemical Advanced Resolution Methods Laboratory, Michigan Technological University, Houghton, MI 49931, USA; mkhaksar@mtu.edu; 4 Alliance to Feed the Earth in Disasters (ALLFED), Fairbanks, AK 99775, USA; david@allfed.info or ddenkenberger@alaska.edu; 5 University of Alaska Fairbanks, Fairbanks, AK 99775, USA

**Keywords:** alternative food, edible leaves, edible plants, existential risk, global catastrophic risk, leaf, leaf concentrate, leaf protein, non-target screening, public health, sustainable food systems, toxins

## Abstract

Alternative food supplies could maintain humanity despite sun-blocking global catastrophic risks (GCRs) that eliminate conventional agriculture. A promising alternative food is making leaf concentrate. However, the edibility of tree leaves is largely uncertain. To overcome this challenge, this study provides the methods for obtaining rapid toxics screening of common leaf concentrates. The investigation begins with a non-targeted approach using an ultra-high-resolution hybrid ion trap orbitrap mass spectrometer with electrospray ionization (ESI) coupled to an ultra-high pressure two-dimensional liquid chromatograph system on the most common North American leaf: the red maple. Identified chemicals from this non-targeted approach are then cross-referenced with the OpenFoodTox database to identify toxic chemicals. Identified toxins are then screened for formula validation and evaluated for risk as a food. The results after screening show that red maple leaf concentrate contains at least eight toxic chemicals, which upon analysis do not present substantial risks unless consumed in abundance. This indicates that red maple leaf is still a potential alternative food. The results are discussed in the context of expanding the analysis with open science and using leaf extract from other plants that are not traditionally used as foods to offset current global hunger challenges, and move to a more sustainable food system while also preparing for GCRs.

## 1. Introduction

Several low (but non-zero) probability global catastrophes could partially block the sun and render conventional agriculture incapable of preventing mass human starvation [1]. Of these risks the most probable sun-obscuring scenario is nuclear war with the burning of cities (nuclear winter) [2,3]. Two quantitative models indicate that the probability of full-scale nuclear war is at approximately 1% per annum [4,5]. Yet the majority of nuclear powers possess more than the pragmatic limit of nuclear weapons [6]. The pragmatic limit is set at where the direct physical negative consequences of nuclear weapons use are counter to national interests. Either a one-sided relatively minor nuclear weapons use on target population centers [6] or a small regional nuclear war [7,8] could render a global “nuclear autumn”, which would starve millions [6,7,8,9,10]. Similarly, an asteroid or comet impact [11], as well as a super volcanic eruption or continental basalt flows could induce the same long-term sunlight deprivation and mass starvation as conventional agriculture fails [12,13,14]. In addition, several less-severe risks to the agricultural system could aggravate food accessibility globally including: (i) abrupt climate change [15]; (ii) extirpating crop pathogens [16]; (iii) super weeds [17]; (iv) super crop pests [18]; (v) super bacterium [19]; (vi) coincident extreme weather, resulting in multiple breadbasket failures [20]; (vii) slow climate change that is extreme (>~5 °C) [21]; or (viii) pollinator loss [22]. Other catastrophes would not directly affect food production, but still could have similar impacts on human nutrition. Some of these include a pandemic or non-nuclear world war that disrupts global food trade, and the resultant famine caused in food-importing countries [23,24,25]. Though some of these catastrophes affect other needs, such as water and energy, the most difficult problem historically is provision of food. Avoiding such global catastrophe risks (GCRs) would be in the best interest of all humans on the planet, yet even for the GCR for which humanity has complete control (nuclear war), it appears unlikely to be abated in the short term and as of this writing there are not proven reliable means for avoiding the other GCRs and considerable further research is needed in this area [26,27,28,29]. These agricultural GCRs rank high on the list of GCRs in terms of impact and probability [30]. In summary a sustainable food system demands preparation for GCRs.

It is; thus, rational to have some form of backup source of food available. The most obvious would be storing up food ahead of time, but this would take years, would increase the price, exacerbate current malnutrition, and would be very expensive [31]. Previous work has determined that *alternative food* supplies could be viable in GCRs that eliminate all conventional agriculture [32]. Specifically, the entire human population could be maintained based on caloric intake, even in the extreme global catastrophes that block sunlight for five years, by converting fossil fuels, dead trees (wood), and leaves to human-edible food [1,32]. Preliminary calculations even show that by eating a variety of these alternative foods it is possible to obtain a balanced diet of macronutrients [32] and micronutrients [33] to maintain reasonable human health [34]. Preparing for alternative food production is a very cost-effective way to save expected lives, both globally [35] and from the perspective of only the United States [36]. These calculations are all based on technical feasibility and considerable future research is necessary for more extreme scenarios [37,38], particularly considering the transition from conventional to alternative foods and being able to afford the food without subsidy [39]. It is critical to have a resilient global food system [40] that is continuous (e.g., able to maintain caloric intake consistently). The largest challenge with this transition is between the time that stored foods run out globally following a sun-blocking global catastrophe (about six months) [1,32] and the time that many alternative foods are ramped up to full production (about one year) [1,32]. The best theoretical solution for this transition problem is to use leaves killed by the catastrophe (as opposed to leaves that are depleted of nutrients and shed naturally (leaf litter)), especially because the price compared to many alternative foods is reasonable [39]. However, food from leaves presents several challenges [32]. First a small fraction of the calories can be extracted. For example, with black tea, only about 20% of the total calories of the proteins, carbohydrates, and lipids make it into the liquid [41]. Eating tree leaf-based teas is also uncommon (although many people already drink pine needle tea [42]). In addition, it is possible to grind and press the leaves, and then coagulating the resultant liquid as leaf concentrate, which contains ~8% of the dry matter of the original leaves [43]. The remaining liquid contains much of the toxins, and has been considered unfit for human consumption [44]. The yield of the leaf concentrate is lower with nonindustrial techniques, which would be more widely accessible [44]. Unfortunately, there is a distinct lack of knowledge on the toxins and their quantities found in the most common tree leaves and the ability of humans to consume leaf extract from common tree leaf types.

Therefore, this study attempts to provide the preliminary steps for obtaining a rapid toxics screening process of common leaf concentrates to be used for alternative foods. The combination of high-resolution mass spectrometry (HRMS) and chromatography techniques has initiated a new pathway for detection and identification of unknown compounds in complex mixtures [45]. The traditional approach for detection of compounds in a solution is to do targeted screening by purchasing the corresponding reference standards for identification [45]. In targeted screening, the information regarding the mass, structure, isotopic pattern, and retention time are known and used for detection and quantification purposes. For the potential toxic compounds in plant materials this presents both an egregiously time-consuming and expensive process, as there are over 1500 phytotoxins already identified in the Toxic Plants–Phytotoxin (TPPT) Database [46]. In recent years, the evolution of accurate mass HRMS has allowed for non-targeted screenings where no prior information is available for identification of unknowns in a sample [45]. In non-targeted screening, evidence from the measurement data is needed to confirm the identification using a suitable algorithm. This evidence includes accurate mass (within an acceptable error tolerance), isotope pattern, presence of additional adducts, retention time, fragmentation information, and other experimental evidence. The amount of evidence available for identification leads one to various levels of confidence for compound identification [47,48], which have been discussed in detail [45]. The investigation begins with a non-targeted approach using an ultra-high-resolution hybrid ion trap orbitrap mass spectrometer (MS) coupled to an ultra-high-pressure two-dimensional liquid chromatograph (LC) system on the most common leaf in North America. Of the more than 800 different tree species in the U.S. [49], red maple (*acer rubrum*) is the most common [50]. Thus, for the U.S. it provides the largest quantity of potential alternative food calories for any tree species. Red maple leaf concentrate is fabricated using small-scale processing equipment, which is widely accessible and analyzed using ThermoFisher Scientific’s screening method of unwanted compounds in food [51]. These identified chemicals from this non-targeted approach are then cross-referenced among several databases to identify toxic chemicals and any other potential harmful effects (e.g., if they are mutagenic, teratogenic, carcinogenic, etc.). The results on red maple leaves are presented and the data is shared using an open science method to make further analysis of the dataset possible. The toxic chemicals identified are then screened for risk to humans. The results are discussed and future work is determined in the context of open data and using leaf extract to offset current global hunger challenges and sustainable food systems while preparing for GCRs.

## 2. Materials and Methods 

### 2.1. Materials

#### 2.1.1. Leaf Selection

Yellow maple leaves have been eaten as a snack in tempura form in Japan, known as “momiji,” [52] so there is the potential that this highly common leaf could be a source of alternative food. However, the veterinary literature warns that red maple is toxic to horses [53]. There is; thus, a need to screen it for potential toxicity to humans. The red color of maple leaves can be used to distinguish it from other maple tree species. Red maples are deciduous and the leaves, which are typically 5–10 cm long and wide with 3–5 palmate lobes with a serrated margin, are arranged oppositely on the twig. The upper side of the red maple leaf is light green and the underside is whitish and can be either glaucous or hairy. The leaf stalks are usually red and are up to 10 cm long and the leaves generally turn a deep red in the autumn. Red maple trees were identified in Houghton, MI, and collected for processing.

#### 2.1.2. Processing Chemicals

The control was Houghton, MI tap water. Tap water was used to more closely replicate the processing conditions for leaf concentrate in a global catastrophe situation. LC/MS grade acetonitrile and water were purchased from Fisher Scientific (Waltham, MA, USA) and HoneyWell (Morris Plains, NJ, USA), respectively. LC/MS grade formic acid was purchased from Sigma Aldrich (St. Louis, MO, USA).

### 2.2. Material Processing

#### 2.2.1. Leaf Concentrate

Red maple leaves were processed in batches. First, the leaves were blended and wrapped in a cheesecloth. Then the material was placed in rapidly boiling water for five minutes. The leaf fragments were removed with a stainless-steel sieve while pressing the cheesecloth. The skimmed concentrate was then collected in a clean glass container and then analyzed.

#### 2.2.2. Sample Preparation

For liquid chromatography/mass spectrometry (LC/MS) analysis, the red maple leaf extract was diluted 12 times in water–acetonitrile 80:20 (v:v) and filtered through a 0.2 µm quartz filter.

### 2.3. LC/MS Instrumentation 

The Thermo Scientific Dionex Ultimate 3000 standard system was used as high-pressure liquid chromatography (HPLC) system. The analytical column was Phenomenex reversed-phase Kinetex XB-C18, 150 × 2.1 mm, 100 $\dot{A}$, with 1.7 µm particle size. Mobile phase A was 0.1% formic acid in 100% LC/MS grade water and mobile phase B was 0.1% formic acid in LC/MS grade acetonitrile–water 95:5 (v:v) solution. Using a constant flow rate of 0.2 mL/min (0.2 mL/min was used due to small particle size of the column (1.7 um), which increases the back pressure), the mobile phase gradient was: 0 min; 5%B, 5 min; 5%B, 65 min; 90%B, 70 min; 90%B. The column was equilibrated with mobile A for 15 min before the next injection. The column oven was set at 35 °C, and the full loop injection volume was set at 5 μL.

The mass spectrometry instrument was a Thermo Scientific Orbitrap Elite equipped with electrospray ionization (ESI). The resolving power for accurate mass measurement during the LC/MS run was 120 K defined at *m/z* 400. Red maple leaf extract was run with both positive and negative ESI modes under two separate LC/MS runs. The instrument was calibrated externally with Thermo Pierce calibration solution before LC/MS runs. All the masses in the range of 100–600 *m/z* were recorded with full scan mode. In addition to the full scan, data-dependent MS/MS fragmentation was also recorded for the 5 tallest peaks on each spectral scan with a collision energy of 25 (arbitrary unit). This was done to help identify co-eluting compounds.

### 2.4. Data Analysis

The LC/MS results were then analyzed by Thermo Scientific Compound Discoverer software for identification of unknown compounds. Compound Discoverer enables evaluation of mass spectral data, predicts the elemental composition, and searches databases for possible identities of candidate compounds. An automated workflow in Compound Discoverer was created by connecting nodes containing parameters that were adjusted to obtain optimal identification results. The mass tolerance determined for chemical identification was set to 3 ppm and minimum intensity was set to 20,000. The elemental compositions considered over the ranges of C_1–90_H_1–190_O_0–18_N_0–5_S_0–1_, under the conditions of 0.1 ≤ H/C ≤ 3.5, 0 ≤ double-bond equivalent (DBE) ≤ 40. The ChemSpider database was used for parent mass and elemental composition searches. The ChemSpider databases chosen for the experiment were: ActoR, aggregated computation, DrugBank, EPA DSSTOX (Distributed Structure-Searchable Toxicity (DSSTox) Database – developed by the U.S. Environmental Protection Agency), EU-Open Screen, FDA-Unit-NLM (Food and Drug Administration Unit National Library of Medicine), Food and Agriculture Organization, FooDB (maintained in Canada it is the world’s largest and most comprehensive resource on food constituents), LeadScope, Lhasa Limited, Pesticide Common Names, and Wikipedia. Then, Compound Discoverer uses isotopic pattern recognition, fragments, and isotopic distribution to generate confirmation of identity. The data-dependent MS/MS results were used for spectral searches in the mzCloud and Thermo Scientific mzVault environments to confirm compounds structures. The generation of elemental composition uses isotopes, accurate mass, and fragments to generate match scores [49]. If match score is high, the identification of compound is done with high levels of confidence, as discussed above.

**Figure A1:**
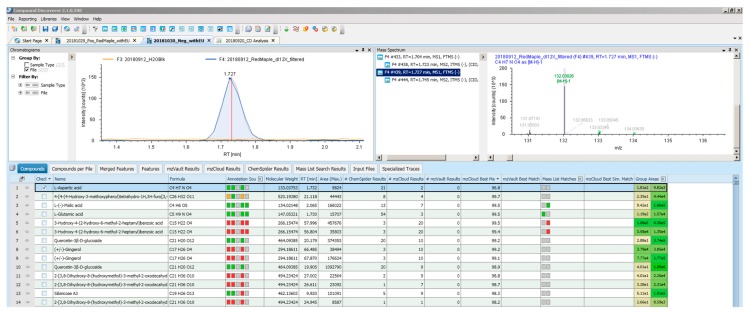
Example of LC/MS chromatogram, mass spectrum, and results table from Compound Discover findings in red maple leaf concentrate.

These chemicals were then cross-referenced in the European Food Safety Authority’s (EFSA’s) OpenFoodTox database of chemical hazards [54] (creative commons attribution 4.0 international license) for toxicity and any other potential harmful effects (e.g., mutagenic, teratogenic, carcinogenic, etc.). The toxic compound list that resulted was then sent for formula validation with the following steps:

1. Checked to make sure that the compound is only present in the sample and not in the blank (see the extracted ion chromatogram on the top left of Appendix A).
2. Checked that the chromatographic peak is above the noise level. A signal to noise ratio (S/N) of 3 was used in the software and a minimum peak area of 1000 considered to validate the chromatography peaks from the noise.
3. Checked that the chromatographic peak shape is good. This is done by looking at the extracted ion chromatogram generated by Compound Discoverer software for each compound to ensure that the chromatography peak shape is reliable.
4. Checked the isotopic pattern. After Compound Discoverer assigns a chemical formula to the measured masses, it generates color-coded isotopic patterns on the mass spectrum of each compound (see the example mass spectrum associated with L-Aspartic acid on the top right of the Figure A1 in Appendix A).
5. Compound Discoverer software searched ChemSpider databases for possible chemical structures for each assigned formula. The number of chemical structures found in ChemSpider that match the measured mass with the defined ppm mass error (3 ppm) is recorded.
6. Compound Discoverer also searched a mass list developed by Thermo Scientific for leachable and extractable compounds.
7. Finally, MS/MS mzCloud match is determined. With the LC/MS analysis, rather than the full scan mode, the data-dependent MS/MS fragmentation was collected on the 5 tallest peaks on the spectra. Compound Discoverer uses this information to match the fragmentation pattern with the mzCloud database. This adds another level of confidence for identification of unknown compounds.

Finally, the positively identified toxic compounds were further evaluated independently, using the NIH-hosted PubChem open chemistry database, to determine their potential risks if red maple leaf concentrate were used as a food in a catastrophe.

## 3. Results

**Figure 1:**
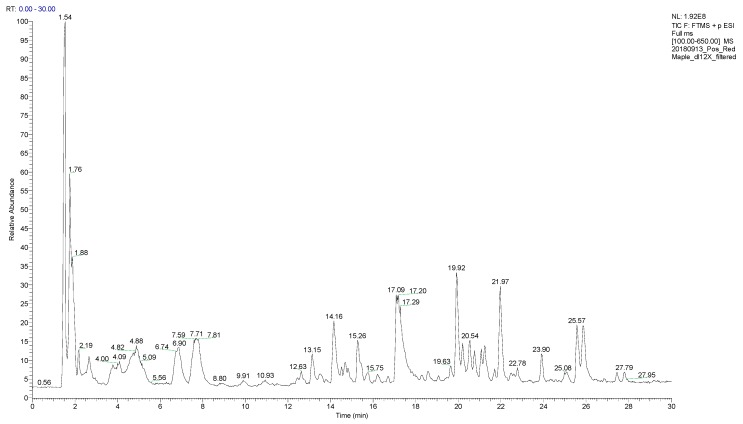
Total ion chromatogram of red maple leaf concentrate with LC/MS, positive ESI, C_18_ Column, with ACN-H_2_O (acetonitrile-water) gradient. No peak was detected after 30 min so the chromatogram was cut at 30 min.

**Figure 2:**
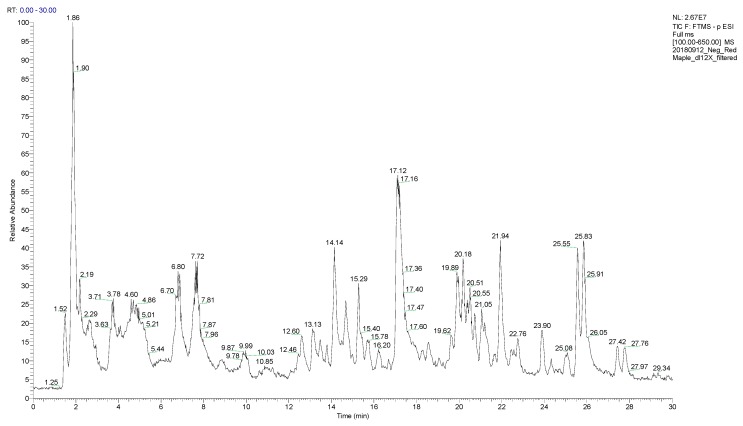
Total ion chromatogram of red maple leaf concentrate with LC/MS, negative ESI, C_18_ Column, with ACN-H_2_O (acetonitrile-water) gradient. No peak was detected after 30 min so the chromatogram was cut at 30 min.

As expected for a leaf (due to the relatively well-documented green tea analysis [55,56]) there are thousands of compounds detected with each ionization mode: positive ESI contained 4380 masses and in negative ESI 1267 masses. Some of those are common between both polarities and some of them are unique to each. The chromatogram for red maple leaf concentrates for positive ESI is shown in Figure 1 and for negative ESI is shown in Figure 2. All of the raw data are available on the Open Science Framework [57]. LC/MS positive and negative ESI results were then analyzed with Compound Discoverer Software to extract the chromatographic peaks and assign chemical formula.

Using the ChemSpider databases listed above (toxic libraries), 31 chemicals were identified with a high level of confidence from the positive ESI and seven from the negative ESI, with three appearing in both (gallic acid, L-glutamic acid, and salicylic acid). This number was further reduced after the formula validation shown in Table 1 and Table 2 for the positive and negative ESI, respectively. As can be seen in Table 1, the positive ESI uncovered five chemicals where there was a high degree of confidence that they are toxic and in the red maple leaf, which included: (1) 3-methoxybenzaldehyde, (2) 4-methoxybenzaldehyde, (3) coumarin, (4) L-glutamic acid, and (5) L-phenylalanine.

As can be seen in Table 2, the negative ESI uncovered four chemicals where there was a high degree of confidence that they are toxic and in the red maple leaf, which included: (1) citric acid, (2) L-aspartic acid, (3) L-glutamic acid, and (4) naringin. L-glutamic acid was found in both positive and negative ESI, so there are a total of eight toxic compounds that may preclude the use of red maple leaf concentrate as a food source.

## 4. Discussion

### 4.1. Do Toxic Compounds Prevent Maple Leaf Concentrate Use as a Food?

The results of this analysis have shown that there are at least eight toxic chemicals in the red maple leaf concentrate, which include: (1) 3-methoxybenzaldehyde, (2) 4-methoxybenzaldehyde, (3) coumarin, (4) L-glutamic acid, (5) L-phenylalanine, (6) citric acid, (7) L-aspartic acid, and (8) naringin. However, with even a cursory analysis of the chemicals, the majority of the eight do not preclude the use of red maple leaf as a candidate alternative food. 3- and 4-methoxybenzaldehyde is a common compound found in fragrances and may cause respiratory tract irritation [58]. L-glutamic acid is one of the 20 proteinogenic amino acids and a key molecule in cellular metabolism [59]. In humans in reasonable amounts it is easily digested [60]. Although ingestion of monosodium glutamate (MSG) can cause transient clinical symptoms resembling those of “Chinese Restaurant Syndrome” and there is some evidence that a minority may respond to relatively small doses [59], it is not a significant risk. Similarly, L-aspartic acid is another non-essential amino acid found in animals and plants, especially in sugar cane and sugar beets [61]. It must be eaten in abundance to become an issue [61]. L-phenylalanine is an essential amino acid in humans, which is provided by eating food and would be viewed as a macronutritional asset, except for a tiny minority suffering from phenylketonurics and some individuals with schizophrenia [62]. Citric acid is a weak acid common in citrus fruits and does not present a danger unless consumed in abundance or inhaled [63]. Similarly, naringin is the major flavonoid glycoside in grapefruit and gives grapefruit juice its bitter taste. It is metabolized in humans and presents a risk only in abundance or by inhalation [64].

Although the majority of the toxic chemicals are not a major issue when using red maple leaf concentrate as a source of food, coumarin is of greater concern because it is considered a poison, although found in many plants which are used as food sources [65]. The use of coumarin as a food additive was banned by the US FDA in 1954 based on reports of hepatotoxicity in rats and because of the hepatotoxic effects in humans, the European Commission (EC) restricted coumarin from naturals as a direct food additive to 2 mg/kg food/day. However, the European Regulation No 1334/2008 EC granted exceptions for higher levels for alcoholic beverages, caramel, chewing gum, and certain “traditional foods”, which included 50 mg/kg in traditional and/or seasonal bakery ware containing a reference to cinnamon in the labeling, 20 mg/kg in breakfast cereals including muesli, 15 mg/kg in fine bakery ware, with the exception of traditional and/or seasonal bakery ware containing a reference to cinnamon in the labeling, and 5 mg/kg in desserts. A recent study found fine bakery ware investigated exceeds the European limit (15 mg/kg) in almost 50% of the cases and that tea can be a significant contributor to overall coumarin intake [66]. Future work is needed to quantify the amount of coumarin in red maple leaf concentrate to determine how serious of a potential source of toxicity it would be in a global catastrophe.

### 4.2. Limitations and the Need for Open Source Collaboration

This study has several limitations in the context of showing that red maple leaf extract is safe to eat, and as such this method would be expected to have the same limitations when extended to other types of leaves to be used as an alternative food. First, it is possible that this leaf concentrate or one of the others could have a toxic chemical in it that has not been studied in the past and; therefore, the method does not prove that it is a safe food. However, even for the toxic chemicals previously determined, this method may miss them because of the type of extraction that was used for preparing the leaf concentrate. Some of the less polar compounds may need to be extracted with organic solvents, while here the water extraction technique was used. In addition, chemicals have several names and thus if the OpenFoodTox database used other names they might be missed by the comparison algorithm. Compound Discoverer is connected to more than 300 libraries through ChemSpider node. The libraries chosen for this study were the ones suggested by Thermo Scientific [67] and suspected to include toxic compounds. More work could be done to test other libraries for other possible harmful compounds. Additionally, the current available software for chemical formula identification can mostly handle CHNOS compounds for reliable assignments. Designing a more appropriate algorithm to include more elemental composition with reliable formula assignment is the task that can be done in the future. In addition, an ultra-high-pressure two-dimensional liquid chromatography system was employed, but the analysis shown here was for mono-dimensional LC-MS method. In the future, research can use another dimension to resolve more peaks and perhaps identify more compounds. The other approach could be to use other MS ionization techniques, such as atmospheric pressure chemical ionization (APCI) [68] and atmospheric pressure photoionization (APPI) [69] for detection of less polar toxic compounds. ESI used in this work is the most widely used ionization technique that covers a large range of molecular weights and polarities; however, some non-polar compounds may not be detectable with ESI and require other ionization techniques. The other limitation of this work is the lack of MS/MS information in the libraries for validation of the chemical structures, which emphasize the need for open source collaborations.

Determining the toxicity of such a complicated potential alternative food is challenging. Compound Discoverer has different workflows that can be chosen for compounds identifications. This study originally tried two different workflows. One of them found 20 compounds with the predicted chemical formula to be within the 3 ppm, which also has a high matching score for data-dependent MS/MS. This means that one can be very confident that these are assigned correctly. With the other workflow, only two compounds with good matching scores were found. It means that different workflows can find different compounds. Thus, unlike in many studies in this field, here the full data set is being made available as open source [57]. Based on previous work that has indicated that science can be accelerated by promoting an open research culture [70,71,72,73] and applying an open source approach to software [74,75,76] and hardware [77,78,79,80,81]. If this method becomes a standard, then other researchers can build upon the initial characterization done by any group to obtain a better understanding of the chemical makeup of a potential alternative food.

Finally, there are a lot of other compounds that do not have data-dependent MS/MS fragmentation because they were not the five tallest peaks on the spectra. Thus, more experiments can be conducted to get MS/MS fragmentation on more peaks (e.g., data-dependent MS/MS on the second five tallest peaks, and so on). A suspect screening can also be conducted to generate MS/MS information on a list of known masses from the toxic libraries [45].

An alternative to identifying toxins individually and assessing their risk is using the idea of “substantial equivalence” [82] used by regulators (such as the U.S. FDA) to approve novel whole foods. This would involve firstly performing compositional analysis and in vitro toxicological tests on a leaf (ideally from related tree leaves, like yellow maple) that has historically been eaten by humans, and then on the tree in question (e.g., red maple). If compositions and toxicological results are similar, then the leaf could be deemed relatively safe for ingestion (even if known toxic compounds are present, or not all compounds can be identified).

### 4.3. Alternative Food in Today’s Non-Disaster Context

The concept of alternative foods was originally developed to provide food for people during a global catastrophe that completely eliminated conventional agricultural production. There are a number of other possible scenarios where alternative food could be relevant. The first scenario is the 10% global agricultural shortfalls that could dramatically increase food prices. Second is local disasters such as earthquakes and hurricanes that could cut off external food supply. Finally, there is the scenario of normal functioning of systems. Even when our agricultural system is in full operation, there are six million preventable deaths per year in children under five years old caused by malnutrition and hunger-related disease [83]. Thus, alternative foods, such as leaves, could be extracted from living trees now in appropriate quantities, to provide for a more sustainable food system while offsetting some malnutrition. There are more than 820 million undernourished people that face chronic food deprivation throughout the world [84]. This unnecessary hunger, suffering, and death is due to an inadequate sharing of the earth’s resources, as there are more than enough calories generated by conventional global agriculture to easily feed everyone on the planet. This makes global hunger more of a problem involving the poor’s inability to acquire food at an acceptably low cost. Alternative food offers the potential for nutrition for the poor at low cost and future work is needed in several areas to probe that potential. First, research is needed to expand the methodology in this study to all of the major leaf types throughout the world in order to take the first steps towards guaranteeing that these foods sources are not toxic. Second, research is needed to evaluate the technical viability of using leaf concentrate in non-catastrophe scenarios. Finally, an economic study is needed to evaluate the costs of leaf concentrate used as a human food source based on geographical location, leaf type, and extraction technology.

## 5. Conclusions

This study provided the first steps of a novel approach for obtaining a rapid toxics screening process of common leaf concentrates to be used for alternative foods. This non-targeted approach is much faster and less costly than conventional methods. The results when applying this approach to the most common tree type in North America found that red maple leaf concentrate contains at least eight toxic chemicals. However, upon analysis based on toxicity data for these chemicals in PubChem, it is clear they do not present substantial risks unless consumed in abundance. This indicates that red maple leaf is still a potential alternative food, although more research is needed on the concentration and impacts of getting a significant fraction of one’s calories from leaf concentrate. There is a clear need for expanding this analysis using open science to other types of leaf extracts, to help offset current global hunger challenges, to move humanity towards a sustainable food system, as well as to better prepare humanity/civilization to survive a low-probability high-impact GCR.

## Figures and Tables

**Table 1:** Formula validation of positive ESI.

Name	Retention Time	Not in the Blank	Above Noise	Good Peak Shape	MS isotopic Pattern	MS Chem Spider Match	Leachable and Extractable Mass List Match	MS/MS mzCloud Match
2,3,6-Trimethylphenol	16.70	✔	✔	✔	[M + H − H_2_O] match	50	5	no match
3-Methoxybenzaldehyde	11.49	✔	✔	✔	[M + H] match	50	5	3 or 4 methoxybenzaldehyde, 82%
4-Methoxybenzaldehyde	12.99	✔	✔	✔	[M + H] match	50	5	3 or 4 methoxybenzaldehyde, 82%
8-Hydroxyquinoline	7.95	✔	✔	✔	[M + H] match	4	1	no MS/MS
Bensulfuron-methyl	1.70	✔	✔	✔	[M + H] match	50	1	no MS/MS
benzaldehyde	23.12	✔	✔	✔	[M + H] match	9	1	no MS/MS
Cinnamaldehyde	13.97	✔	✔	✔	[M + H] match	50	No match	no MS/MS
Coumarin	17.63	✔	✔	✔	[M + H] match	32	No match	80%
Erythorbic acid	2.10	✔	✔	✔	[M + H] match	27	No match	no MS/MS
Diphenylamine	42.50	✖	✔	✔	[M + H] match	50	2	no MS/MS
Ethyl benzoate	19.65	✔	✔	✔	[M + H] match	50	1	no MS/MS
Ethyl cinnamate	21.71	✔	✔	✔	[M + H] match	50	No match	Benzyl Methacrylate, 61%
Gallic acid	13.15	✔	✔	✔	[M + H − H2O] match	16	No match	no MS/MS
Indole	7.95	✔	✔	✔	[M + H] match	42	No match	no MS/MS
L-Glutamic acid	1.74	✔	✔	✔	[M + H] match	50	No match	99.40%
L-Histidine	1.59	✔	✔	✔	[M + H] match	49	1	no MS/MS
L-Isoleucine	2.56	✔	✔	✔	[M + H] match	50	2	no MS/MS
L-Methionine	1.75	✔	✔	✔	[M + H] match	40	1	no match
L-Phenylalanine	4.02	✔	✔	✔	[M + H] match	50	1	99.60%
L-Proline	1.80	✔	✔	✔	[M + H] match	50	No match	no MS/MS
L-Tyrosine	13.37	✔	✔	✔	[M + H] match	50	1	no MS/MS
Naringin dihydrochalcone	22.78	✔	✔	✔	[M + Na] match	0	No match	no match
Nicotinamide	2.12	✔	✔	✔	[M + H] match	49	No match	no MS/MS
Propyl gallate	8.56	✔	✔	✔	[M + H] match	49	1	no MS/MS
salicylic acid	15.77	✔	✔	✔	[M + H] match	39	1	no MS/MS
Tentoxin	19.06	✔	✔	✔	✖	6	No match	no match
Terephthalic acid	9.71	✔	✔	✔	[M + H] match	40	3	no MS/MS
Triphenylphosphine oxide	34.86	✖	✔	✔	[M + H] match	10	1	no MS/MS
Thidiazuron	1.92	✔	✔	✔	[M + H] match	31	1	no MS/MS
Trichlorfon	1.51	✔	✔	✔	[M + H] match	2	1	no MS/MS
Vanillin	16.17	✔	✔	✔	[M + H] match	50	2	no MS/MS

**Table 2:** Formula validation of negative ESI.

Name	Retention Time	Not in Blank	Above Noise	Good Peak Shape	MS isotopic Pattern	MS Chem Spider Match	Leachable and Extractable Mass List Match	MS/MS mzCloud Match
Citric acid	2.46	✔	✔	✖	[M − H] match	12	2	89.4%%
Gallic acid	3.72	✔	✔	✔	[M − H] match	16	no match	no match
L-Aspartic acid	1.73	✔	✔	✔	[M − H] match	21	no match	96.8
L-Glutamic acid	1.73	✔	✔	✔	[M − H] match	50	no match	99.50%
Naringin	22.93	✔	✔	✔	[M − H] match	12	no match	80.30%
Salicylic acid	21.71	✖	✔	✔	[M − H] match	40	1	no MS/MS
Succinic acid	2.77	✔	✔	✔	[M − H] match	16	1	no MS/MS

## Appendix A

The chromatogram on the top left (Figure A1) is related to L-aspartic acid chosen from the results table on the bottom. The blank is shown with orange color and the sample is shown with blue. The peak is only present in the sample and not in the blank. The software has also validated the isotopic pattern of L-aspartic acid on the mass spectrum on the top left. The monoisotopic peak is indicated with lavender, and the isotopic peaks are indicated with green color; if they are present and have correct intensities.
